# Establishment of a prognostic signature and immune infiltration characteristics for uterine corpus endometrial carcinoma based on a disulfidptosis/ferroptosis-associated signature

**DOI:** 10.3389/fimmu.2025.1492541

**Published:** 2025-01-27

**Authors:** Yong Huang, Huibin Li, Zhifu Wei, Wanshan He, Bin Chen, Shuang Cheng, Zhifang Zhao, Lv Deng, Xiaohua Chen, Yu Lin, Xiaoshan Hong

**Affiliations:** ^1^ Department of Gynecology, Guangdong Women and Children Hospital, Guangzhou, China; ^2^ Department of Pathology, Guangdong Women and Children Hospital, Guangzhou, China; ^3^ Department of Gynecology, The Affiliated Shunde Hospital of Jinan University, Foshan, China; ^4^ Department of Gastroenterology, Guizhou Provincial People’s Hospital, Guiyang, China; ^5^ Department of Gastroenterology, People’s Hospital of Rongjiang County, Rongjiang, China; ^6^ Department of Gastroenterology, People’s Hospital of Nanhai District, Foshan, China; ^7^ Oncology Center, Southern Medical University Hospital of Integrated Traditional Chinese and Western Medicine, Southern Medical University, Guangzhou, China; ^8^ Nanfang Hospital, Southern Medical University, Guangzhou, China; ^9^ Department of Gastroenterology, Southern Medical University Hospital of Integrated Traditional Chinese and Western Medicine, Southern Medical University, Guangzhou, China; ^10^ Department of Gynecology, Qingxin District Hospital of Women and Children Healthcare, Qingyuan, China

**Keywords:** UCEC, uterine corpus endometrial carcinoma, disulfidptosis, ferroptosis, prognostic signature, immune infiltration

## Abstract

**Background:**

Disulfidptosis and ferroptosis are two different programmed cell death pathways, and their potential therapeutic targets have important clinical prospects. Although there is an association between the two, the role of genes associated with these two forms of cell death in the development of endometrial cancer remains unclear.

**Methods:**

In this study, RNA sequencing (RNA-seq) and clinical data were obtained from public databases, and comprehensive analysis methods, including difference analysis, univariate Cox regression, and Least Absolute Shrinkage and Selection Operator (LASSO) analysis were used to construct a disulfidptosis/ferroptosis-related genes (DFRGs) prognostic signature. To further explore this new feature, pathway and functional analyses were performed, and the differences in gene mutation frequency and the level of immune cell infiltration between the high- and low-risk groups were studied. Finally, we validated the prognostic gene expression profile in clinical samples.

**Results:**

We identified five optimal DFRGs that were differentially expressed and associated with the prognosis of uterine corpus endometrial carcinoma (UCEC). These genes include CDKN2A, FZD7, LCN2, ACTN4, and MYH10. Based on these DFRGs, we constructed a robust prognostic model with significantly lower overall survival in the high-risk group than in the low-risk group, with differences in tumor burden and immune invasion between the different risk groups. The expression of two key genes, ACTN4 and LCN2, was verified by immunohistochemistry and RT-qPCR.

**Conclusion:**

This study established a clinical prognostic model associated with disulfidptosis/ferroptosis-related genes, and the expression characteristics of key genes were validated in clinical samples. The comprehensive assessment of disulfidptosis and ferroptosis provides new insights to further guide patient clinical management and personalized treatment.

## 1 Introduction

Uterine corpus endometrial carcinoma (UCEC) is a common malignant gynecological disease, and the prevalence rate of UCEC has increased by 132% in the past three decades (1). Currently, the key to the diagnosis of endometrial cancer is the pathological evaluation of endometrial tissue, but this invasive test is only useful for patients who have undergone endometrial biopsy or ultrasound and who exhibit endometrial thickening. Despite treatment guidelines from the European Society of Gynecologic Oncology, which are based on clinical staging and molecular subtyping, there are differences in the prognoses of patients with endometrial cancer according to risk group (2). Approximately 15% of patients with urothelial carcinoma (UCEC) are at an advanced stage at the time of diagnosis. In addition, approximately 15 to 20% of patients are at risk of recurrence after initial surgical treatment (3, 4), and the prognosis for this subset of patients remains poor (5). Research on the early diagnosis of endometrial cancer and its prognostic risk factors is still in its infancy. Currently, no feasible clinical prognostic model has been developed, which limits the effective formulation of personalized treatment strategies. Disulfidptosis is a unique type of cell death pathway identified in recent biological studies (6). Excessive cystine induces rapid apoptosis due to the increase in disulfide bond pressure, which has extraordinary potential value in predicting the end of cells and the effectiveness of immunotherapy. Ferroptosis, a specific form related to iron-dependent cell death, has received extensive attention in the treatment of drug-resistant endometrial cancer in recent years (7–9)and some articles have reported that some ferroptosis-related genes have important value in predicting the prognostic status of endometrial cancer patients (10, 11).

Although disulfidptosis and ferroptosis are two different forms of cell death, they share a common regulator plasma carrier family 7 member11 (SLC7A11). Cancer cells rely on SLC7A11 cystine input to maintain redox balance and cell survival, which can inhibit the occurrence of ferroptosis, and SLC7A11 downregulation and methylation can induce ferroptosis in endometrial cancer cells (12, 13), however, when the SLC7A11 gene is overexpressed and glucose deprivation occurs, disulfidptosis death is triggered. Therefore, this exploratory work is the first to link disulfidptosis with ferroptosis. By analyzing large-scale public data resources, we screened genes related to these two phenomena (disulfidptosis/ferroptosis-related genes, (DFRGs)) and established a prognostic model of their coexistence. This model provides a comprehensive perspective for understanding the composite effects of DFRGs on UCEC. After careful screening, we identified five genes associated with survival probability (CDKN2A, FZD7, LCN2, ACTN4, and MYH10) as useful biomarkers for disease identification and prognostic assessment. Based on the risk ratings of these genes, we subdivided UCEC patients into several different prognostic classes. We subsequently compared the differences between the higher- and lower-risk categories in terms of immune score, and immune cell penetration, and initially confirmed them in actual clinical tissue samples. The aim of this study was to determine the feasibility of a personalized medical protocol for UCEC patients, with the expectation of optimizing their postoperative recovery prospects.

## 2 Materials and methods

### 2.1 Dataset information

RNA sequencing data (HTSeq-FPKM) and clinical prognostic information on UCEC patients were obtained from the TGCA database (https://portal.gdc.cancer.gov/). A total of 589 cases were included in the analysis, including 554 endometrial cancer samples and 35 normal tissue samples. (**Supplementary Figure S1**). Out of these, 560 cases included clinical information. we implemented the following preprocessing steps to ensure data quality: (1) Cases with a reported survival time of zero or those lacking survival time information were excluded from our analysis, resulting in the removal of 16 cases. (2) In instances of missing clinical data other than survival time, we applied appropriate imputation methods where feasible or documented exclusions to maintain data integrity and transparency.

We screened 512 ferroptosis-associated genes (FAGs) through a comprehensive review of the FerrDb database (14) (http://zhounan.org/ferrdb/legacy/index.html, July 1, 2023), which is a curated resource focused on genes and molecules associated with ferroptosis. The identification of the disulfidptosis-related genes mainly referenced the protein-protein interaction network of the proteins with disulfide bonds, reported by Liu et al. (15). We applied a filter to include only those genes that had at least two supporting studies cited in the literature. This led to the inclusion of the following key genes: NADPH, INF2, SLC7A11, CD2AP, DLIM1, MYH9, ACTN4, IQGAP1, MYH10, FLNB, FLNA, MYL6, TLN1, DSTN, CAPZB and ACTB.

### 2.2 Differential analysis of disulfidptosis-related and ferroptosis-associated genes

In this study, a preliminary collation of the RNA-seq dataset and associated clinical information was performed to eliminate incomplete records. First, the “Limma” R software package was used to screen the differentially expressed disulfidptosis/ferroptosis-related genes (DE-DFRGs) using the criteria of |log2FC| > 0.5 and FDR < 0.05. This selection was made to capture potential candidate genes that may play a role in cancer prognosis to the greatest extent possible, balancing sensitivity and specificity. The correlation between disulfidptosis and ferroptosis was calculated via the Spearman method using the “psych” R software, and a Holm-adjusted significance test was performed with P value correction to generate a network showing the association between the two.

### 2.3 Construction of the DFRG prognostic model and validation

To explore the DE-DFRGs associated with disease prognosis, we first partitioned the dataset into a training set (70%) and a validation set (30%). In the training set, we performed Cox proportional hazard model analysis on the independent variables and identified differentially expressed genes that were considered significantly associated with the survival period. Subsequently, Least Absolute Shrinkage and Selection Operator (LASSO)-Cox regression analysis was performed to filter out genes with strong collinearity, aiming to identify genes that are critical for prognosis. Specifically, we performed variance inflation factor (VIF) analysis prior to applying LASSO-Cox regression, predictors with VIF values greater than 5 were considered indicative of multicollinearity and were either removed or combined where appropriate. Additionally, we have included details on the optimization of the regularization parameter using 10-fold cross-validation, which helps to minimize the overfitting risk and determine the most appropriate lambda value. The predictive models were established using the “GLMnet” and “Survival” packages in R software. The prognostic risk score of the TCGA-UCEC cohort was estimated using the “Model Predictions” R package, and patients were classified into different prognostic classes according to the median risk score (low -risk or high -risk). The Kaplan-Meier method, stratified Cox regression, multivariate Cox regression, and ROC curve analysis were used to confirm the validity and reliability of the prognostic model. Calibration curves were generated via a canonical graph model to produce a prognostic graph depicting the survival of patients with endometrial cancer at 1-, 3-, and 5- years. Additionally, we conducted external validation using two independent transcriptomic datasets from the GEO database (GSE21882 and GSE115810). Applying our prognostic model to these cohorts, we compared risk factors associated with different 5-year survival outcomes and tumor differentiation levels.

### 2.4 GO, KEGG and mi-RNA regulatory relationship

Gene ontology (GO) and Kyoto Encyclopedia (KEGG) enrichment analyses were used to further explore the biological processes and pathways associated with the DFRGs, via ‘Cluster Profiler’ R software (version 3.14.3). Due to the high number of tests performed in enrichment analysis, we utilized the Benjamini-Hochberg (BH) method to control the false discovery rate (FDR), thereby improving the reliability of the findings. In addition, the UCEC miRNA sequence data were obtained from the TCGA database. The “Limma” package of R was used to explore the differences in miRNA expression. Prognostic miRNAs were screened via a univariate Cox regression model. We employed Pearson or Spearman correlation coefficients to assess the relationships between miRNAs and their target genes, depending on the distribution of the data.

### 2.5 Tumor mutational burden analysis

After the analysis, single nucleotide polymorphism (SNP) information for UCEC was extracted from the TCGA database, which is helpful for exploring the associations between prognosis and cancer. Through the “maftools” R software package, the TMB value of each patient was calculated. Only non-synonymous variants in coding regions were considered for the TMB calculation, as these are more likely to have functional implications in tumor biology. Previous studies have shown that using the median is a common practice in TMB analysis, ensuring that our approach aligns with established conventions in the field. Accordingly, all patients were subsequently divided into two groups based on the median TMB value: one group included samples with high TMB values, and the other included samples with low TMB values. We identified the top variant genes with significant differences when high and low -risk categories were compared and explored the correlation between the TMB and risk score.

### 2.6 Immune infiltration of the disulfidptosis/ferroptosis-related prognostic model

We obtained the abundance of infiltrating immune cells through various algorithms adopted by the TIMER 2.0 (http://timer.cistrome.Org/) database. To account for potential batch effects and noise in the immune infiltration data, we utilized the ComBat method from the “sva” package in R, which adjusts for batch effects in the data. Twenty-two immune cell infiltrates between different risk groups were evaluated via the CIBERSORT algorithm, and the results were visualized via the “vioplot” R package. The prognostic differences between different immune infiltration profiles were subsequently analyzed.

### 2.7 Expression levels of key prognostic genes in biological samples

In this study, we collected postoperative biological samples and paraffin-fixed sections from patients treated at Guangdong Women and Children’s Hospital from January 2023 to August 2023. The patients signed a consent form before surgery for the donation of their excised tissue, which was stored in our pathology laboratory, for scientific research. After the model was constructed, this study submitted a clinical sample use application to the Ethics Committee of Guangdong Women and Children Hospital according to standard procedures and received ethical approval (Approval No. 202301078). Five endometrial cancer tissue samples and sections were approved for key gene validation, one of which was used for preexperiments (clinical information is detailed in Appendix File 2).

During the sample application process, the samples were reviewed by pathology experts and labeled as tumor tissue or adjacent tissue. Fresh tissue indicated a sample retained for rapid pathological testing during surgery and was stored in liquid nitrogen, a -80°C environment or a dry ice transport box until it was used for detection. The mRNA expression levels of CDKN2A, FZD7, LCN2, ACTN4 and MYH10 were quantitatively analyzed via real-time quantitative polymerase chain reaction (RT−qPCR). We specified that all experiments were performed in triplicate and included information on the assessment of technical variability. Each sample was analyzed in at least three independent biological replicates to account for biological variability. we employed the ΔΔCt method for relative quantification of gene expression. The statistical methods used to analyze the RT-qPCR results included a paired t-test for comparing expression levels between tumor and adjacent normal tissues when analyzing matched samples.

RNA was extracted from the tumor samples and normal tissues via TRIzol reagent (Ambion, USA). A quantitative reverse transcription kit (Promega, USA) was subsequently used to reverse transcribe the products into cDNA. Quantitative PCR (qPCR) is a technique for measuring the DNA content of a sample in real time. The SYBR-Green reagent of the Vazyme Company was used to carry out real-time fluorescence quantitative qPCR detection, and the expression level of each sample was corrected and standardized to the expression level of actin. The primers used are detailed in Appendix S3.

All the slides were processed by first incubating them at 60°C for 20 minutes, deparaffinizing them in xylene, and rehydrating them in gradient ethanol. After incubation with 3% hydrogen peroxide for 10 min, the slides were soaked in 0.01 M citrate buffer (pH 6.0) to recover the antigen for 30 min. The slides were incubated overnight at 4°C with their corresponding antibodies after being blocked with 5% bovine serum albumin. Then, another biotinylated antibody was added at room temperature for one hour. The progress of immunohistochemical staining was monitored by using diaminobenzidine as the reaction medium, and restraining was performed with hematoxylin.

### 2.8 Statistical analysis

In this study, we adopted a series of analytical validation methods for bioinformatics and statistical analysis. Differentially expressed gene screening and model construction are primarily performed using R software (4.2.0), which is derived from publicly available versions in CRAN and Bioconductor. For differential expression analysis, we utilized the DESeq2 package, using the DESeq() function to normalize and analyze RNA-seq data. For survival analysis, the survival package was used, specifically the coxph() function to fit Cox proportional hazards models, and analysis of survival curves was conducted using the survfit() function. For LASSO-Cox regression, we implemented the glmnet package, utilizing the cv.glmnet() function for cross-validation and lambda optimization. For immune infiltration analysis with CIBERSORT, we utilized custom R scripts compatible with the CIBERSORT algorithm to process RNA-seq data. Enrichment analysis for GO and KEGG was performed using the clusterProfiler package with functions such as enrichGO and enrichKEGG. The t test and Wilcoxon rank sum test were performed to analyze the differences between two sample groups, whereas the Kruskal−Wallis test was used to analyze the differences between two or more sample groups. We set the threshold for statistical significance to p<0.05, and all data analysis was performed via R software.

## 3 Results

### 3.1 Differential expression of disulfidptosis-related and ferroptosis-associated genes in UCEC

**Figure 1:**
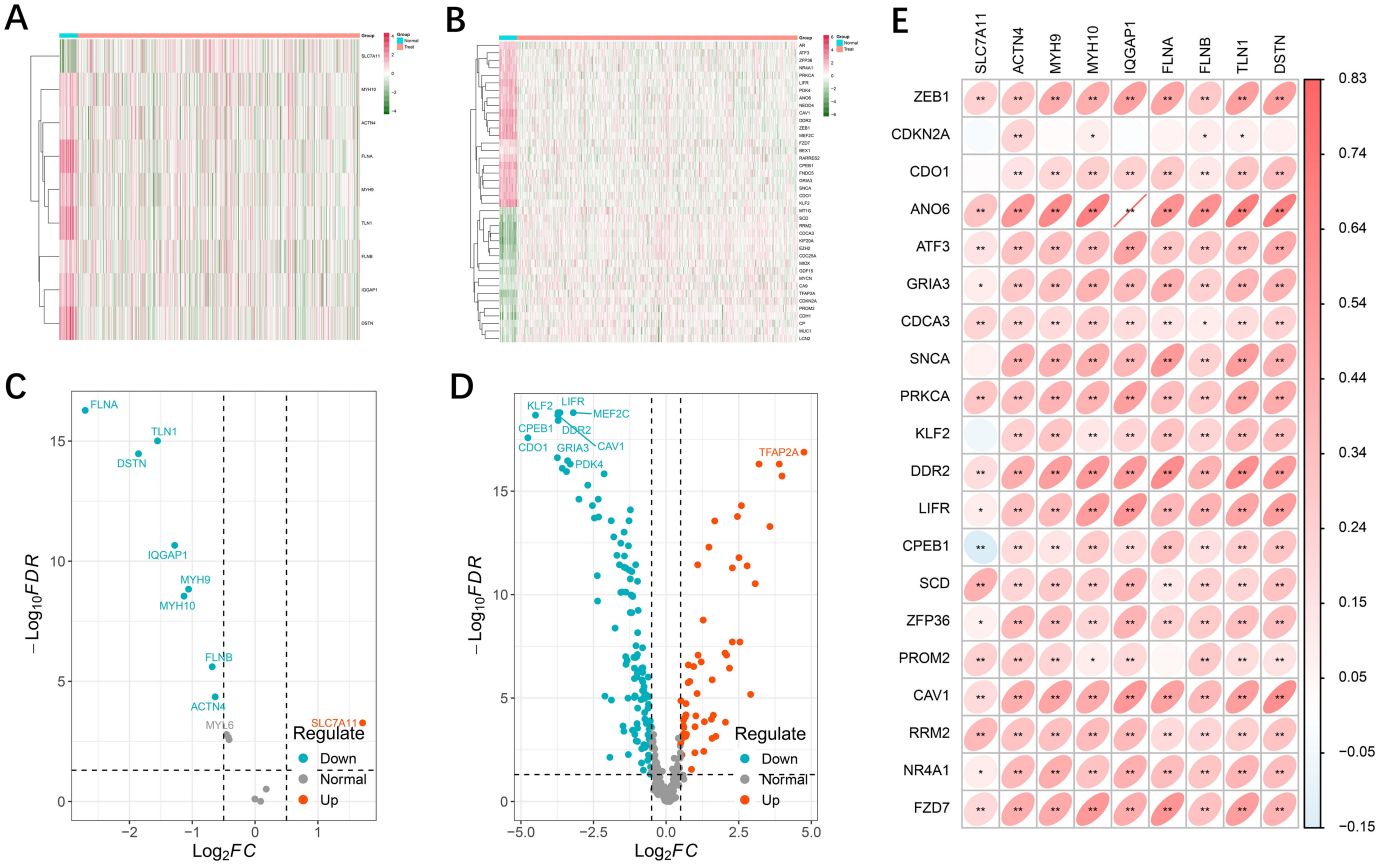
Differential expression of disulfidptosis- and ferroptosis-related genes. **(A, B)** Heatmap showing the disulfidptosis- and ferroptosis-related genes in tumor and normal adjacent tissues. Green represents downregulation, and red represents upregulation of the gene. **(C, D)** Volcano map of 9 differential disulfidptosis-related genes and 178 differential ferroptosis-related genes. **(E)** Interactive correlation heatmap between differential disulfidptosis-related genes and ferroptosis-related genes (p <0.01 = **, and p < 0.05 = *).

We analyzed the differential expression of 16 disulfidptosis-related genes and 512 ferroptosis-related genes in the TCGA-UCEC cohort (**Supplementary Figure S1**). DE-DFRGs were identified via the “limma” R package, and expression heatmaps were generated for normal versus tumor tissues (**Figures 1A, B**). Finally, 10 genes associated with disulfidptosis (**Figure 1C**) and 41 genes associated with ferroptosis (**Figure 1D**) were screened and mapped to volcanoes. In addition, we explored the correlation between the differential genes related to disulfidptosis and ferroptosis and found that there was a significant positive correlation between the expression of disulfidptosis genes and the expression of most ferroptosis genes (**Figure 1E**, Appendix File 4).

### 3.2 Establishment of the DFRG model

**Figure 2:**
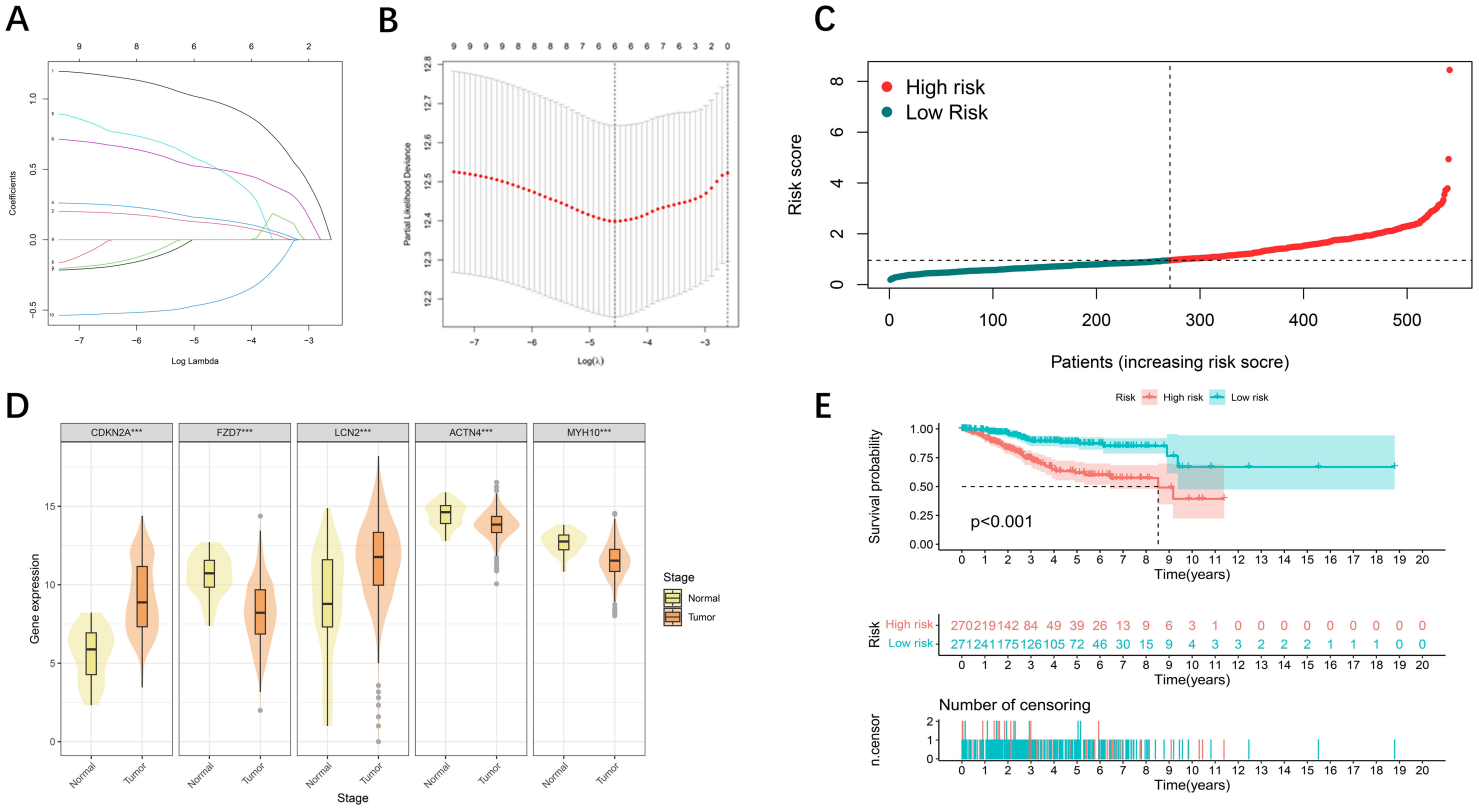
Development of prognostic features of the DE-DFRG model. **(A, B)** Lasso-Cox regression analysis revealed 5 DE-DFRGs. **(C)** Risk score curve graph. The green curves indicate the low-risk group, and the red curves indicate the high-risk group. **(D)** Expression of 5 DE-DFRGs between the UCEC samples and normal samples ( ***p < 0.001). **(E)** Kaplan-Meier survival curves. Survival time was shorter in the high-risk group in the TCGA-UCEC cohort.

To further screen DFRG genes related to survival prognosis, we first performed univariate Cox regression on the differential genes associated with disulfidptosis and ferroptosis, calculated the relationships between the changes in the expression of individual genes and the prognostic characteristics of patients (**Supplementary Figure S2**), followed by further screening of the common differential genes via a LASSO regression model (**Figures 2A, B**). Finally, a prognostic risk model based on five genes was constructed with the following formula: risk score = [CDKN2A expression × (1.3412)] + [FZD7 expression × (0.7441)] + [LCN2 expression × (-0.5069)] + [ACTN4 × (0.7265)] + [MYH10 expression × (-0.7140)]. Using the median risk score as a criterion for differentiation, we subdivided UCEC patients into high- and low-risk groups (**Figure 2C**). The statistical distributions of the five different DE-DFRG expression patterns are illustrated below (**Figure 2D**). The data from the KM curve revealed that overall survival was significantly lower in the high-risk group than in the low-risk group (**Figure 2E**).

### 3.3 Validation of model accuracy

**Figure 3:**
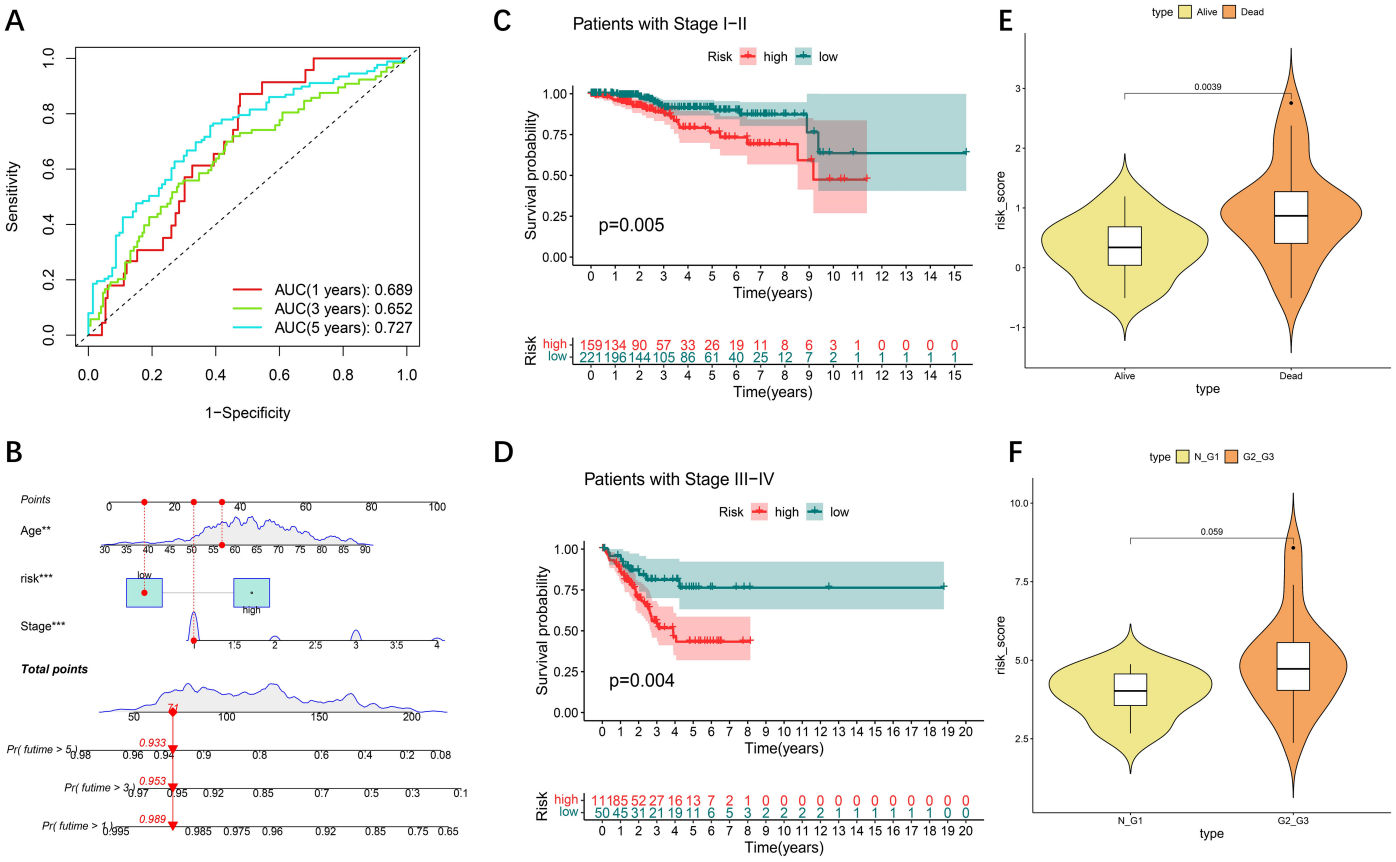
Internal validation of the DE-DFRG model by TCGA-UCEC. **(A)** Time-dependent ROC curve and AUC of the prognostic signature in UCEC patients from TCGA. **(B)** Nomogram for the prediction of 1-, 3-, and 5-year overall survival. **(C, D)** Kaplan-Meier survival curves of patients with clinical stage I-II and III-IV disease. **(E)** Violin plot of risk scores between patients who survived for five years and those who did not. **(F)** Violin plot of risk scores between normal/G1 stage tumors to G2/G3 stage tumors.

Time-based ROC curve analysis was performed to determine the predictive efficacy of the signal, and the area under the operating characteristic curve (AUC) of the observed object was 0.689 at 365 days; at 1905 and 1825 days, the values were 0.652 and 0.727, respectively (**Figure 3A**). We then constructed a model for predicting one-, three-, and five-year survival in patients with UCEC based on relevant prognostic indicators, which showed significant accuracy in predicting the outcome (**Figure 3B**). We evaluated the risk signature regard to pathological features (tumor stage), high risk was significantly associated with more severe stages (**Figures 3C, D**). We included two additional analyses to further validate our prognostic model. we compared the risk scores for patients those who survived to five years with those who did not (GSE21882 from GEO dataset), and the risk scores between normal/G1 stage tumors with G2/G3 stage tumors (GSE115810 from GEO dataset). The results indicated that patients with poorer 5-year survival outcomes (**Figure 3E**) and lower tumor differentiation (**Figure 3F**) had significantly higher risk scores.

### 3.4 Functional enrichment analysis and correlation analysis of microRNA

**Figure 4:**
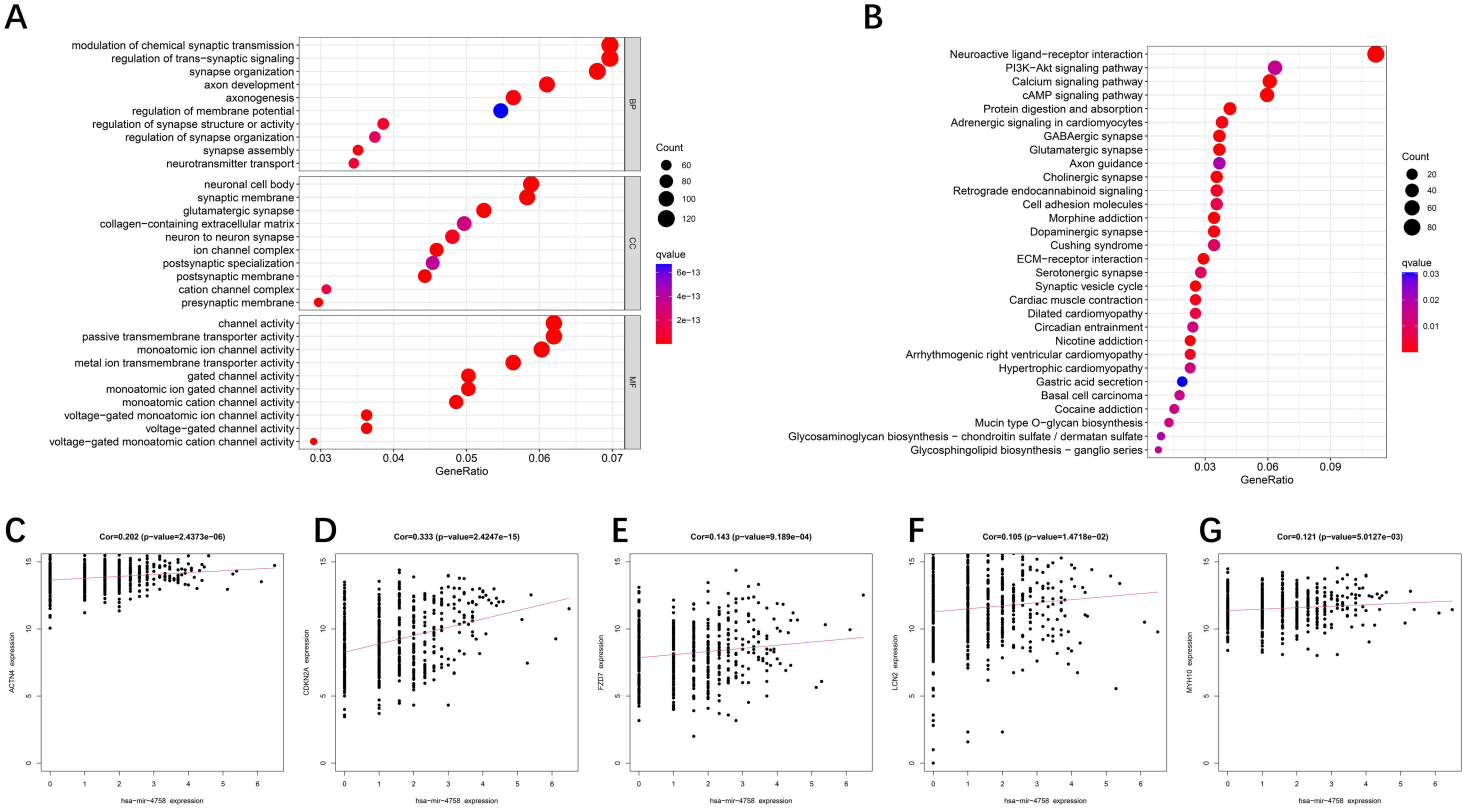
GO/KEGG enrichment analysis of DE-DFRGs and miRNA correlation analysis. **(A, B)** GO and KEGG functional enrichment analyses of the differentially expressed genes. **(C–G)** Relationships between miRNAs and the expression of 5 DE-DFRGs in the model.

To explore the hidden biological attributes related to risk assessment indicators, this study analyzed the KEGG and GO functions of differentially expressed genes (DEGs) in high- and low-risk categories. The results revealed that the pathways involved in glycosphingolipid biosynthesis, extracellular mechanism interactions, and signaling are diverse and complex (**Figures 4A, B**). In addition, through deep mining of the TCGA-UCEC RNA-Seq sequence data, we identified a total of 16,877 microRNAs (miRNAs) and analyzed them via univariate Cox regression models to confirm whether these miRNAs are associated with the survival risk of patients with UCEC (**Supplementary Figure S3**). An exploration of the associations between the expression of key genes and miRNAs associated with survival revealed that hsa−mir−4758 was significantly associated with five key genes (**Figures 4C–G**).

### 3.5 Relationship between the tumor mutational burden and the risk model

**Figure 5:**
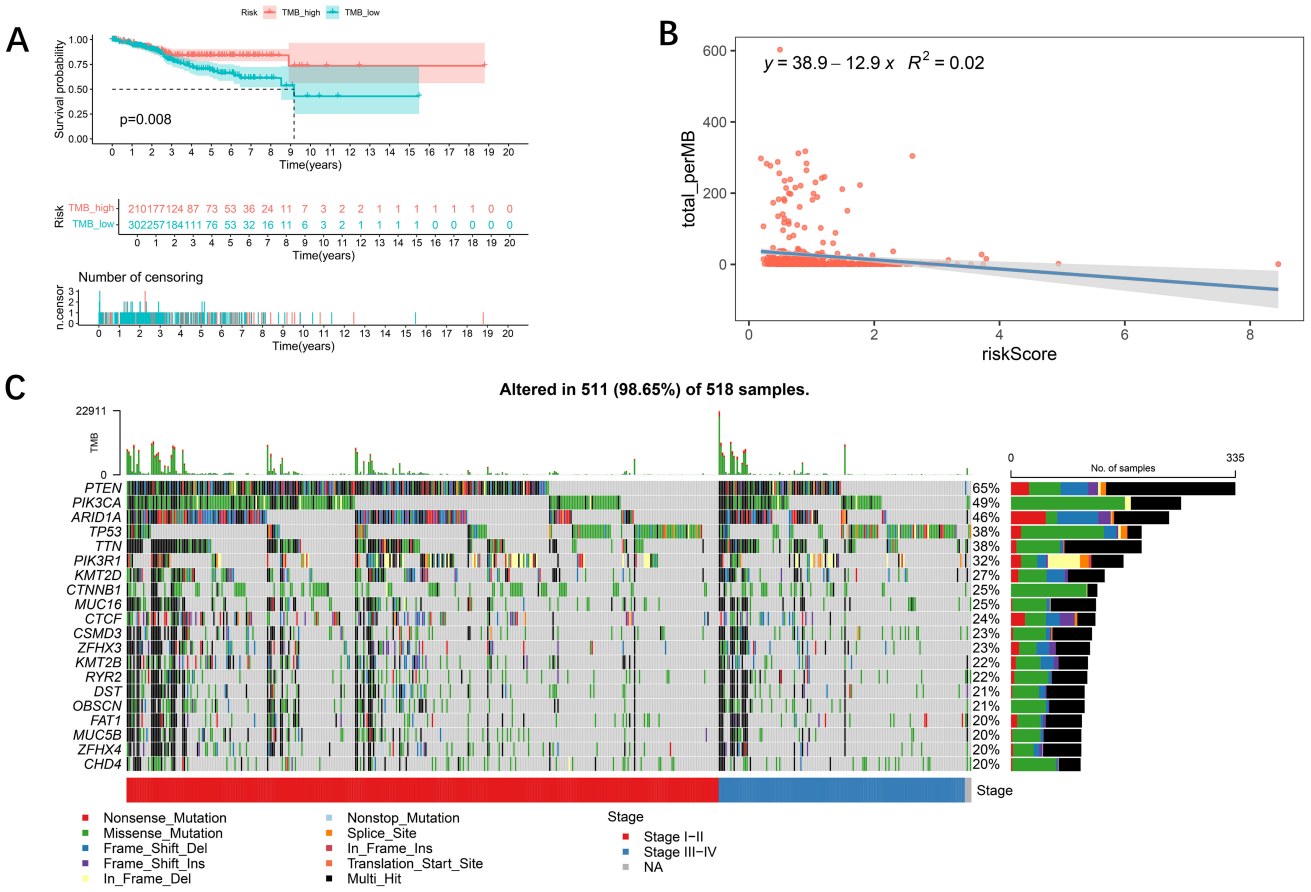
Relationships between the tumor mutational burden (TMB) and the risk model. **(A)** K−M analysis showing the difference in overall survival between the low- and high-TMB groups. In addition, patients with a high TMB had a better prognosis (p < 0.05). **(B)** Relationships between TMB and the risk score. TMB was negatively associated with the risk score (R = −12.9, R2 = 0.02). **(C)** Waterfall plot showing the mutation information on the top 20 genes in each UCEC sample.

The genetic variant density of a tumor, defined as the number of variants found per megabase (MutS per MB), has been associated with the quality of life and prognosis of cancer patients. High levels of tumor genetic variant density (TMB-H) indicate that patients may benefit more from immunotherapy. To more thoroughly explore the role of the risk-prognosis model in predicting the extent of tumor progression, this study explored the association between the model and TMB. First, we performed a Kaplan-Meier analysis on survival data from patients with varying TMB levels and found that patients with higher TMB exhibited better survival rates (**Figure 5A**), which aligns with current perspectives in the field. Subsequently, we analyzed the correlation between TMB and risk scores, revealing that lower risk scores were associated with higher mutation rates (**Figure 5B**), indicating a better prognosis. **Figure 5C** illustrates the differences in gene mutation characteristics between low-risk and high-risk groups, suggesting that variations at the gene level may be key factors contributing to differing prognoses:PTEN (65%), PIK3CA (49%), ARID1A (46%), TP53 (38%) and TTN (38%) were the top five mutated genes.

### 3.6 Signaling-related immune infiltration and LMRG-FAG based immune response

**Figure 6:**
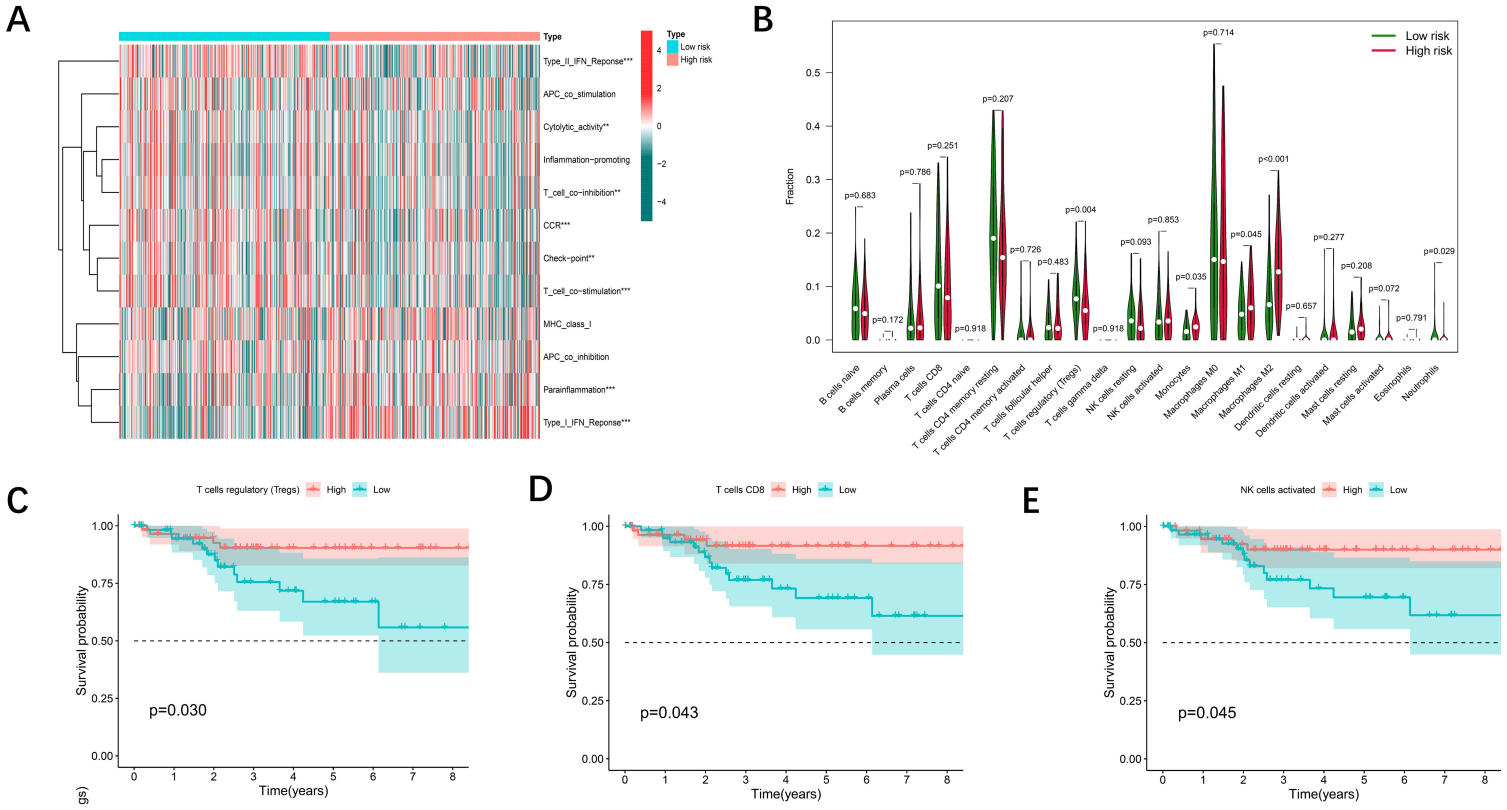
Immune cell infiltration associated with the DE-DFRG model. **(A)** Heatmap showing the comparison of immune-related functions in the high- and low-risk groups. **(B)** The differences in 22 infiltrating immune cells in the TCGA-UCEC cohort between the high- and low-risk groups were analyzed via the Wilcoxon test (p < 0.05). **(C-E)** Kaplan-Meier survival curves illustrating the differences in overall survival between the low and high immune cell infiltration groups.

Recent studies have revealed two key structural changes in the tumor microenvironment, the dissociation of disulfidptosis and ferroptosis, which are closely related to the immune response of tumors. Using the CIBERSORT algorithm tool, we aimed to estimate the distribution of twenty-two immune cell classes in patients with UCEC (**Figure 6A**), and the Violin plot revealed significant differences in the expression levels of regulatory T cells (Tregs), monocytes, macrophages, and neutrophils in different risk classes (**Figure 6B**). Thereafter, we calculated the infiltration scores for 22 types of immune cells and grouped the UCEC patients based on the median scores. A Kaplan-Meier analysis was performed in conjunction with survival data, and the results indicated that higher immune infiltration scores were associated with better patient prognosis (**Figures 6C–E**). Significant heterogeneity in patient survival was observed in the infiltration of several types of immune cells (NK cells, CD8+ T cells, regulatory T cells and highly invasive cells).

### 3.7 Immunohistochemistry and RT-qPCR validation of key prognostic genes

**Figure 7:**
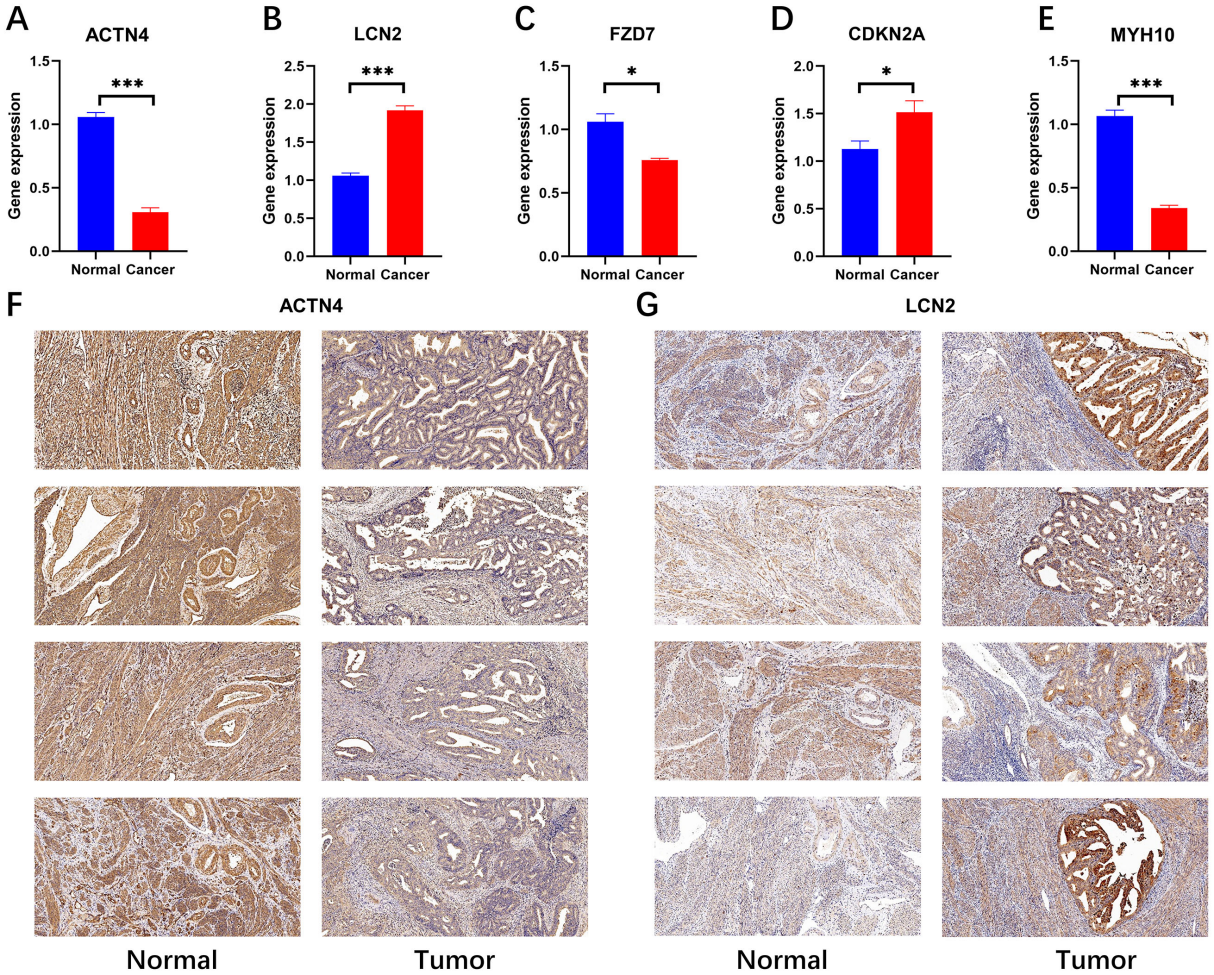
RT−qPCR (N=4) validation and immunohistochemical validation (N=4, x10) of key prognostic genes. **(A-E)** RT−qPCR results revealed that ACTN4, FZD7 and MYH10 were expressed at low levels in cancer tissues and that LCN2 and CDKN2A were highly expressed in cancer tissues (*p < 0.05, and ***p < 0.001). **(F, G)** Immunohistochemical results revealed that ACTN4 was expressed at low levels in cancer tissues and that LCN2 was highly expressed in cancer tissues.

We subsequently confirmed the expression of prognostically relevant genes in clinical samples via real-time quantitative reverse transcription polymerase chain reaction (RT−qPCR) and immunohistochemistry. The experimental data revealed the expression levels of several genes in tumor samples via real-time quantitative polymerase chain reaction (RT−qPCR). Specifically, ACTN4, FZD7, and MYH10 were expressed at low levels in these tumors, whereas LCN2 and CDKN2A were expressed at high levels (**Figures 7A–E**). This finding is in accordance with transcriptional data from The Cancer Genome Atlas (TCGA) based on urothelial cancer (UCEC) samples. The results revealed that the expression of ACTN4 and LCN2 was positive in the cytoplasm and partly in the nucleus and that the expression level of ACTN4 was low (**Figure 7F**), whereas the expression level of LCN2 was high in tumor tissue (**Figure 7G**). This finding was also confirmed by real-time quantitative polymerase chain reaction (RT−qPCR) data. (Photos with higher magnification are detailed in Appendix File 5).

## 4 Discussion

UCEC is a common malignant disease of female reproductive organs, and its prevalence has increased in recent years. For patients, the precise determination of disease stage has a significant effect on the treatment efficacy and patient prognosis. For the first time, the International Federation of Gynecology and Obstetrics (FIGO) has included molecular subtypes in its revised staging guidelines for endometrial cancer in 2023, aiming to provide a more accurate assessment of the prognosis of patients with UCEC. Therefore, the study of new molecular markers and cell communication pathways will provide key theoretical support for interdisciplinary treatment strategies for endometrial cancer. Currently, several ferroptosis-related models have been developed to predict the prognosis of UCEC patients, which are based on a single variable and do not fully consider the complexity of the tumor microenvironment (16, 17). Recent findings indicate a close relationship between ferroptosis and disulfidptosis. Disulfidptosis and ferroptosis are two forms of regulated cell death that share some biochemical pathways and mechanisms. Both processes involve the accumulation of reactive oxygen species (ROS) and perturbations in cellular redox homeostasis, leading to oxidative damage. Recent studies suggest that disulfidptosis may act in concert with ferroptosis, where the oxidative stress from disulfide accumulation can potentially promote ferroptosis (18). In our exploration, we comprehensively considered the correlation between disulfidptosis and ferroptosis. First, we revealed ferroptosis-disulfidptosis differential genes by dissecting gene transcription in tumor and normal tissues. The Lasso algorithm was subsequently applied to filter out the most influential predictor variables, and we constructed a prognostic assessment model consisting of five key genes. Through the analysis of Kaplan−Meier curves, ROC curves and calibration curves the stability, accuracy and reliability of the model were verified.

Recent studies have revealed a strong association between disulfide metabolism and tumor development. In fact, many tumor cells are subjected to oxidative stress, which causes abnormalities in disulfide metabolism, thereby significantly affecting the survival and proliferation of cancer cells (19, 20). The metabolic activity of tumor cells, especially the formation of disulfide bonds, is associated with various biological characteristics of tumors, such as resistance to antibiotics, tumor spread, and immune system evasion (21, 22). Recent studies have shown that specific long noncoding RNAs associated with disulfide dysregulation can provide significant prognostic information for UCEC patients (23). Ferroptosis is caused by the combined effects of intracellular oxidative stress and membrane lipid peroxidation, which leads to the destruction of cells and the termination of life activities. Ferroptosis-related genes and long noncoding RNA coding genes in endometrial carcinomatous tissues are often mutated and epigenetically modulated in contrast to those in conventional tissues (9), resulting in their differential expression in regulating the viability, migration, and invasion of endometrial cancer cells (7, 12). Although a direct association between these genes and endometrial cancer has not yet been established, ferroptosis gene markers play a key role in assessing the prognosis of endometrial cancer patients and formulating targeted treatments. To deepen the research in the field of cancer therapy, explore the interaction between disulfidptosis and ferroptosis signal transduction pathways, reveal the internal relationship between them, and translate this insight into innovative strategies for anticancer therapy, we must build a closer bridge between them.

A recent study revealed that UCEC is one of the two cancer types with the highest mutation rates in disulfidptosis-related genes, whereas mutations in the ACTN4 and MYH10 genes are closely linked to poor survival (18). In the actin-binding protein family, the ACTN4 protein, which was described by Honda and other researchers in the early stage and is considered a nonmuscle α-actinin closely linked to the migration ability of tumor cells, has attracted much attention (24). ACTN4 influences the cell cycle and cell motility and plays a key role in the development and spread of cancer (25), and some studies have shown that ACTN4 behaves differently in endometriotic lesions than in the normal endometrium (26). The MYH10 gene is responsible for the production of nonmuscle myosin IIB (NMIIB), a protein that plays a crucial role in cell adhesion. It also plays a key role in many tumor processes, including promoting cancer cell migration, invasion of surrounding tissues, synthesis of the extracellular matrix (ECM), and triggering the transformation of epithelial cells into mesenchymal cells. CDKN2A is considered to play a key clinical role in assessing various prognostic mechanisms of endometrial cancer (16, 27, 28), possibly related to cell cycle dysregulation caused by CDKN2A deletion (29). Lipocarin 2 (LCN2), a new member of the lipocarin family, is closely related to immune function. Studies have shown that the expression level of LCN2 is increased in many malignant tumors. including lung cancer, breast cancer, prostate cancer, pancreatic cancer and esophageal cancer. Moreover, studies indicate that LCN2 induced ferroptosis is closely related to tumor progression (30). LCN2 serum levels play an important role in the diagnosis of endometrial cancer (31), and upregulation of LCN2 expression promotes drug resistance in endometrial cancer cells and inhibits the ferroptosis process (32). One of the receptors of Wnt signaling, Frizzled 7 (FZD7), plays a key role in the canonical and atypical Wnt pathways. Abnormal activation of the Wnt/β-catenin signaling pathway is closely related to endometrial hyperplasia and its related carcinogenesis (33), and plays a vital role in promoting the spread and migration of tumor cells. Studies have revealed that FZD7 gene expression often increases when ovarian cancer tissues are resistant to platinum-based drugs (34). This increased gene expression increases the sensitivity of tumor cells to ferroptosis.

MicroRNAs(miRNAs) have been shown to coordinate various biological processes and diseases, including the occurrence and progression of cancer (35). Research has shown that miRNAs not only control mRNA expression but also target long noncoding RNAs (36). In recent years, abnormal expression of miRNAs has been reported to be associated with the occurrence of various cancers, and abnormal expression of miRNAs can also induce different epigenetic changes. After screening key prognostic genes, our study further identified hsa-mir-4758 as co-associated with disulfidptosis/ferroptosis-related genes. A comprehensive transcriptome analysis revealed that hsa-mir-4758 is associated with hormone-dependent cancer risk (37). A miRNA-based prognostic model for endometrial cancer also suggested that high expression of hsa-mir-4758 in EC tissue is associated with poor prognosis and a lower survival rate in patients with endometrial cancer (38). This finding may suggest the significant potential value of hsa-mir-4758 in the occurrence and prognosis of endometrial cancer (39).

The characteristic of disulfidptosis is the accumulation of disulfide bonds and the subsequent cellular stress. Cancer cells undergoing disulfidptosis can release a variety of cytoplasmic contents and inflammatory factors, triggering a robust immune response by promoting immune cell infiltration and reprogramming the immunosuppressive tumor microenvironment (TME). This process can activate immune cells and facilitate the occurrence of inflammatory responses (40). On the other hand, ferroptosis is primarily induced by lipid peroxidation caused by iron overload, during which reactive oxygen species (ROS) are generated that can also influence immune cell function. This may alter the recruitment and activation of immune cells within the tumor microenvironment (41). Different forms of regulated cell death can modulate immune infiltration in the tumor microenvironment, thereby affecting therapeutic efficacy against tumors and the overall progression of cancer (42). Immune cells play crucial roles in the stroma of tumors, and they have a significant impact on tumor progression and prognosis (43). Therefore, we performed an in-depth analysis of the immune cell composition in patients with different prognostic grades. The performance of regulatory T cells (Tregs), monocytes, macrophages, and neutrophils varies significantly across risk classes. These observations suggest that patients at lower risk may be more sensitive to immunotherapy, suggesting that they may benefit more from immunotherapy.

However, it must be recognized that our study has inherent limitations. First, our data are based on transcriptome data, so there remains a considerable journey ahead from our research conclusions to their application in a clinical setting. Specifically, understanding how risk stratification derived from our signature could influence the selection between standard therapies and novel treatments requires substantial evidence-based research. Follow-up studies (both prospective and retrospective) are essential to confirm the accuracy of our model. In addition, we need to pay attention to the consistency of samples of various ethnic groups. Moreover, the results of the analysis of the tumor mutation burden and immune invasion profile were only from the TCGA, and more prospective experimental data are needed to confirm these conclusions. Finally, further exploration of the molecular mechanism of UCEC-related risk genes is necessary to explore effective therapeutic targets.

Nonetheless, we believe that our study holds significant value in the exploration of prognosis in endometrial cancer. It underscores the importance of collaboration among oncologists, pathologists, and bioinformaticians to effectively implement our prognostic features in clinical practice. To this end, we are committed to conducting further research at both the foundational and clinical levels to validate our findings and enhance their applicability in real-world settings.

## 5 Conclusions

In summary, by selecting five genes associated with disulfidptosis/ferroptosis-related genes, we created a predictive model of the clinical outcome for UCEC patients based on the TCGA database and validated it internally. In addition, this study explored the mutation burden and immune cell infiltration level of tumors in different prognostic risk groups, which provided possible biomarkers and preliminary evidence for the development of personalized treatment.

## Data Availability

The original contributions presented in the study are included in the article/**Supplementary Materials**, further inquiries can be directed to the corresponding author/s.

## References

[1] Gu B Shang X Yan M Li X Wang W Wang Q. Variations in incidence and mortality rates of endometrial cancer at the global, regional, and national levels 1990-2019. Gynecol Oncol. (2021) 161:573–80. doi: 10.1016/j.ygyno.2021.01.036 33551200

[2] Concin N Matias-Guiu X Vergote I Cibula D Mirza MR Marnitz S. ESGO/ESTRO/ESP guidelines for the management of patients with endometrial carcinoma. Int J Gynecol Cancer. (2021) 31:12–39. doi: 10.1136/ijgc-2020-002230 33397713

[3] Crosbie EJ Kitson SJ McAlpine JN Mukhopadhyay A Powell ME Singh N. Endometrial cancer. Lancet. (2022) 399:1412–28. doi: 10.1016/S0140-6736(22)00323-3 35397864

[4] Lu KH Broaddus RR. Endometrial cancer. N Engl J Med. (2020) 383:2053–64. doi: 10.1056/NEJMra1514010 33207095

[5] Connor EV Rose PG. Management strategies for recurrent endometrial cancer. Expert Rev Anticancer Ther. (2018) 18:873–85. doi: 10.1080/14737140.2018.1491311 29972650

[6] Zheng P Zhou C Ding Y Duan S. Disulfidptosis: a new target for metabolic cancer therapy. J Exp Clin Cancer Res. (2023) 42:103. doi: 10.1186/s13046-023-02675-4 37101248 PMC10134647

[7] Wang Y Wang C Guan X Ma Y Zhang S Li F. PRMT3-mediated arginine methylation of METTL14 promotes Malignant progression and treatment resistance in endometrial carcinoma. Adv Sci (Weinh). (2023) 10:e2303812. doi: 10.1002/advs.202303812 37973560 PMC10754120

[8] Wang Z Shu W Zhao R Liu Y Wang H. Sodium butyrate induces ferroptosis in endometrial cancer cells via the RBM3/SLC7A11 axis. Apoptosis. (2023) 28:1168–83. doi: 10.1007/s10495-023-01850-4 37170022

[9] Zalyte E. Ferroptosis, metabolic rewiring, and endometrial cancer. Int J Mol Sci. (2023) 25. doi: 10.3390/ijms25010075 PMC10778781 38203246

[10] Liu S Zhang Q Liu W Huang X. Prediction of prognosis in patients with endometrial carcinoma and immune microenvironment estimation based on ferroptosis-related genes. Front Mol Biosci. (2022) 9:916689. doi: 10.3389/fmolb.2022.916689 35911966 PMC9334791

[11] Qin J Shao X Wu L Du H. Identification of the ferroptosis-associated gene signature to predict the prognostic status of endometrial carcinoma patients. Comput Math Methods Med. (2021) 2021:9954370. doi: 10.1155/2021/9954370 34531924 PMC8440105

[12] Chen SJ Zhang J Zhou T Rao SS Li Q Xiao LY. Epigenetically upregulated NSUN2 confers ferroptosis resistance in endometrial cancer via m(5)C modification of SLC7A11 mRNA. Redox Biol. (2024) 69:102975. doi: 10.1016/j.redox.2023.102975 38042059 PMC10711489

[13] Murakami H Hayashi M Terada S Ohmichi M. Medroxyprogesterone acetate-resistant endometrial cancer cells are susceptible to ferroptosis inducers. Life Sci. (2023) 325:121753. doi: 10.1016/j.lfs.2023.121753 37160245

[14] Zhou N Yuan X Du Q Zhang Z Shi X Bao J. FerrDb V2: update of the manually curated database of ferroptosis regulators and ferroptosis-disease associations. Nucleic Acids Res. (2023) 51:D571–d582. doi: 10.1093/nar/gkac935 36305834 PMC9825716

[15] Liu X Nie L Zhang Y Yan Y Wang C Colic M. Actin cytoskeleton vulnerability to disulfide stress mediates disulfidptosis. Nat Cell Biol. (2023) 25:404–14. doi: 10.1038/s41556-023-01091-2 PMC10027392 36747082

[16] Jin W Zhuang X Lin Y Zhao X. Integrating ferroptosis-related genes (FRGs) and prognostic models to enhance UCEC outcome prediction and therapeutic insights. J Appl Genet. (2023) 64:723–35. doi: 10.1007/s13353-023-00779-3 37626211

[17] Weijiao Y Fuchun L Mengjie C Xiaoqing Q Hao L Yuan L. Immune infiltration and a ferroptosis-associated gene signature for predicting the prognosis of patients with endometrial cancer. Aging (Albany NY). (2021) 13:16713–32. doi: 10.18632/aging.203190 PMC8266342 34170849

[18] Liu H Tang T. Pan-cancer genetic analysis of disulfidptosis-related gene set. Cancer Genet. (2023) 278-279:91–103. doi: 10.1016/j.cancergen.2023.10.001 37879141

[19] Daly EB Wind T Jiang XM Sun L Hogg PJ. Secretion of phosphoglycerate kinase from tumour cells is controlled by oxygen-sensing hydroxylases. Biochim Biophys Acta. (2004) 1691:17–22. doi: 10.1016/j.bbamcr.2003.11.004 15053920

[20] Hogg PJ. Biological regulation through protein disulfide bond cleavage. Redox Rep. (2002) 7:71–7. doi: 10.1179/135100002125000299 12189052

[21] Chen C Shen M Liao H Guo Q Fu H Yu J. A paclitaxel and microRNA-124 coloaded stepped cleavable nanosystem against triple negative breast cancer. J Nanobiotechnology. (2021) 19:55. doi: 10.1186/s12951-021-00800-z 33632232 PMC7905927

[22] Wang Y Jiang Y Wei D Singh P Yu Y Lee T. Nanoparticle-mediated convection-enhanced delivery of a DNA intercalator to gliomas circumvents temozolomide resistance. Nat BioMed Eng. (2021) 5:1048–58. doi: 10.1038/s41551-021-00728-7 PMC8497438 34045730

[23] Li B Li X Ma M Wang Q Shi J Wu C. Analysis of long non-coding RNAs associated with disulfidptosis for prognostic signature and immunotherapy response in uterine corpus endometrial carcinoma. Sci Rep. (2023) 13:22220. doi: 10.1038/s41598-023-49750-6 38097686 PMC10721879

[24] Honda K Yamada T Endo R Ino Y Gotoh M Tsuda H. Actinin-4, a novel actin-bundling protein associated with cell motility and cancer invasion. J Cell Biol. (1998) 140:1383–93. doi: 10.1083/jcb.140.6.1383 PMC2132673 9508771

[25] Tentler D Lomert E Novitskaya K Barlev NA. Role of ACTN4 in tumorigenesis, metastasis, and EMT. Cells. (2019) 8. doi: 10.3390/cells8111427 PMC6912194 31766144

[26] Honda H Barrueto FF Gogusev J Im DD Morin PJ. Serial analysis of gene expression reveals differential expression between endometriosis and normal endometrium. Possible roles for AXL and SHC1 in the pathogenesis of endometriosis. Reprod Biol Endocrinol. (2008) 6:59. doi: 10.1186/1477-7827-6-59 19055724 PMC2615013

[27] Fang F Wang P Huang H Ye M Liu X Li Q. m(6)A RNA methylation regulator-based signature for prognostic prediction and its potential immunological role in uterine corpus endometrial carcinoma. BMC Cancer. (2022) 22:1364. doi: 10.1186/s12885-022-10490-x 36581816 PMC9801604

[28] Wang Y Ren F Chen P Liu S Song Z Ma X. Identification of a six-gene signature with prognostic value for patients with endometrial carcinoma. Cancer Med. (2018) 7:5632–42. doi: 10.1002/cam4.2018.7.issue-11 PMC6247034 30306731

[29] Kreuger IZM Slieker RC van Groningen T van Doorn R. Therapeutic strategies for targeting CDKN2A loss in melanoma. J Invest Dermatol. (2023) 143:18–25.e11. doi: 10.1016/j.jid.2022.07.016 36123181

[30] Wang D Li X Jiao D Cai Y Qian L Shen Y. LCN2 secreted by tissue-infiltrating neutrophils induces the ferroptosis and wasting of adipose and muscle tissues in lung cancer cachexia. J Hematol Oncol. (2023) 16:30. doi: 10.1186/s13045-023-01429-1 36973755 PMC10044814

[31] Cymbaluk-Ploska A Chudecka-Glaz A Pius-Sadowska E Machalinski B Sompolska-Rzechula A Kwiatkowski S. The role of lipocalin-2 serum levels in the diagnostics of endometrial cancer. Cancer biomark. (2019) 24:315–24. doi: 10.3233/CBM-181942 PMC6484256 30829613

[32] Jiang J Zhu J Qiu P Ni J Zhu W Wang X. HNRNPA2B1-mediated m6A modification of FOXM1 promotes drug resistance and inhibits ferroptosis in endometrial cancer via regulation of LCN2. Funct Integr Genomics. (2023) 24:3. doi: 10.1007/s10142-023-01279-7 38091112

[33] Lan S Zhang Z Li Q. FZD7: A potential biomarker for endometriosis. Med (Baltimore). (2023) 102:e35406. doi: 10.1097/MD.0000000000035406 PMC10553041 37800830

[34] Wang Y Zhao G Condello S Huang H Cardenas H Tanner EJ. Frizzled-7 identifies platinum-tolerant ovarian cancer cells susceptible to ferroptosis. Cancer Res. (2021) 81:384–99. doi: 10.1158/0008-5472.CAN-20-1488 PMC7855035 33172933

[35] Liu H Wan J Chu J. Long non-coding RNAs and endometrial cancer. BioMed Pharmacother. (2019) 119:109396. doi: 10.1016/j.biopha.2019.109396 31505425

[36] Shetty A Venkatesh T Kabbekodu SP Tsutsumi R Suresh PS. LncRNA-miRNA-mRNA regulatory axes in endometrial cancer: a comprehensive overview. Arch Gynecol Obstet. (2022) 306:1431–47. doi: 10.1007/s00404-022-06423-5 35182183

[37] Jayarathna DK Renteria ME Malik A Sauret E Batra J Gandhi NS. Integrative transcriptome-wide analyses uncover novel risk-associated microRNAs in hormone-dependent cancers. Front Genet. (2021) 12:716236. doi: 10.3389/fgene.2021.716236 34512726 PMC8427606

[38] Ni L Tang C Wang Y Wan J Charles MG Zhang Z. Construction of a miRNA-based nomogram model to predict the prognosis of endometrial cancer. J Pers Med. (2022) 12. doi: 10.3390/jpm12071154 PMC9318842 35887651

[39] Wu S Cai W Li Y Tan W Yuan Y Zhou Z. SNHG3/hsa-miR-455-5p axis-mediated high expression of MTHFD2 correlates with tumor immune infiltration and endometrial carcinoma progression. Int J Med Sci. (2023) 20:1097–113. doi: 10.7150/ijms.81962 PMC10357439 37484807

[40] Jin XK Zhang SK Zhang SM Liang JL Yan X Lin YT. Disrupting intracellular homeostasis by copper-based nanoinducer with multiple enzyme-mimicking activities to induce disulfidptosis-enhanced pyroptosis for tumor immunotherapy. Adv Mater. (2024) 37(1):e2410957. doi: 10.1002/adma.202410957 39468892

[41] Gao W Wang X Zhou Y Wang X Yu Y. Autophagy, ferroptosis, pyroptosis, and necroptosis in tumor immunotherapy. Signal Transduct Target Ther. (2022) 7:196. doi: 10.1038/s41392-022-01046-3 35725836 PMC9208265

[42] Jin M Ni D Cai J Yang J. Identification and validation of immunity- and disulfidptosis-related genes signature for predicting prognosis in ovarian cancer. Heliyon. (2024) 10:e32273. doi: 10.1016/j.heliyon.2024.e32273 38952356 PMC11215265

[43] Hinshaw DC Shevde LA. The tumor microenvironment innovately modulates cancer progression. Cancer Res. (2019) 79(18):4557–66. doi: 10.1158/0008-5472.CAN-18-3962 PMC6744958 31350295

