# Supplementary material for: Establishment of a prognostic signature and immune infiltration characteristics for uterine corpus endometrial carcinoma based on a disulfidptosis/ferroptosis-associated signature
Source: Front Immunol. 2025 Jan 27;16:1492541. doi: 10.3389/fimmu.2025.1492541 (PMC11808162; doi:10.3389/fimmu.2025.1492541)

# SUPPLEMENTAL FIGURES

## Supplemental Figure 1: PCA of TCGA-UCEC gene expression levels


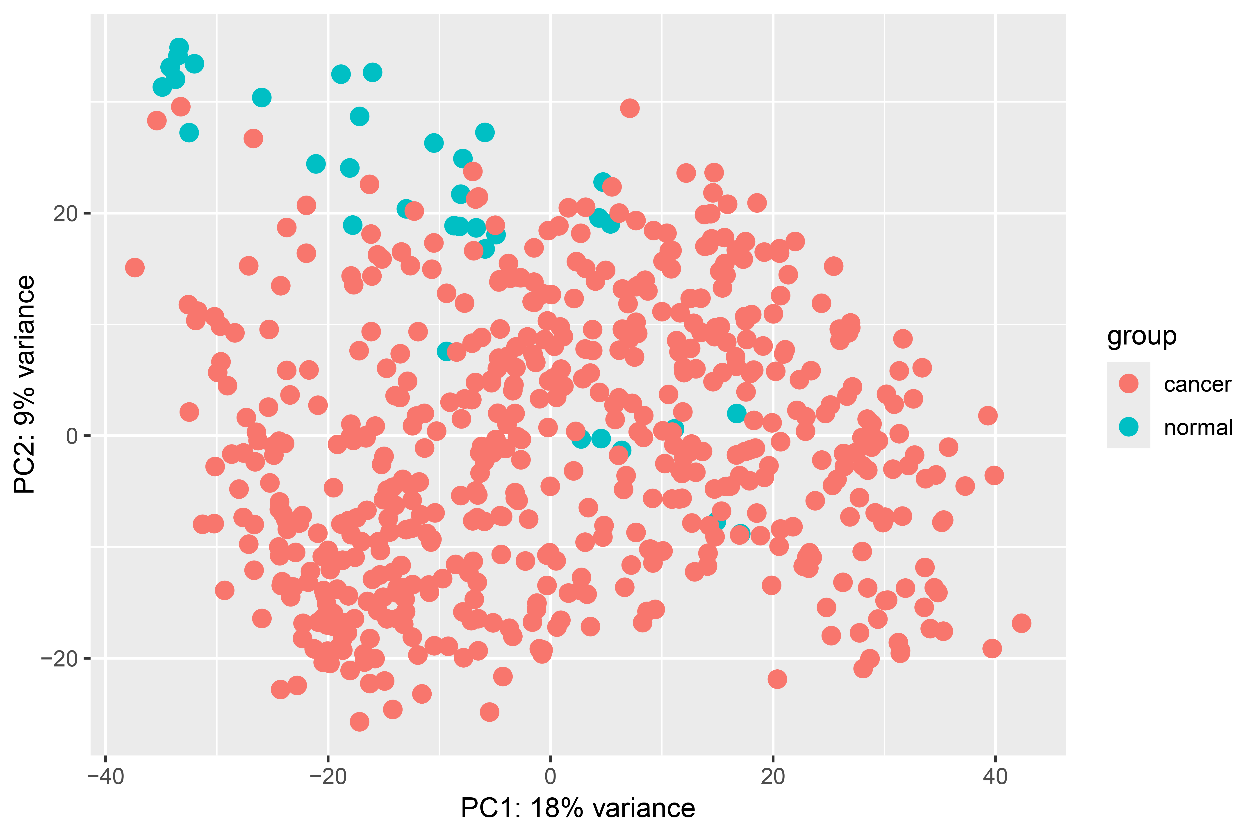


Using R package “DESeq2” for PCA of TCGA-UCEC gene expression levels. Normal group =35, Cancer group = 544.

## Supplemental Figure 2：The KM curves for the gene high-expression and low-expression groups in the construction of the model


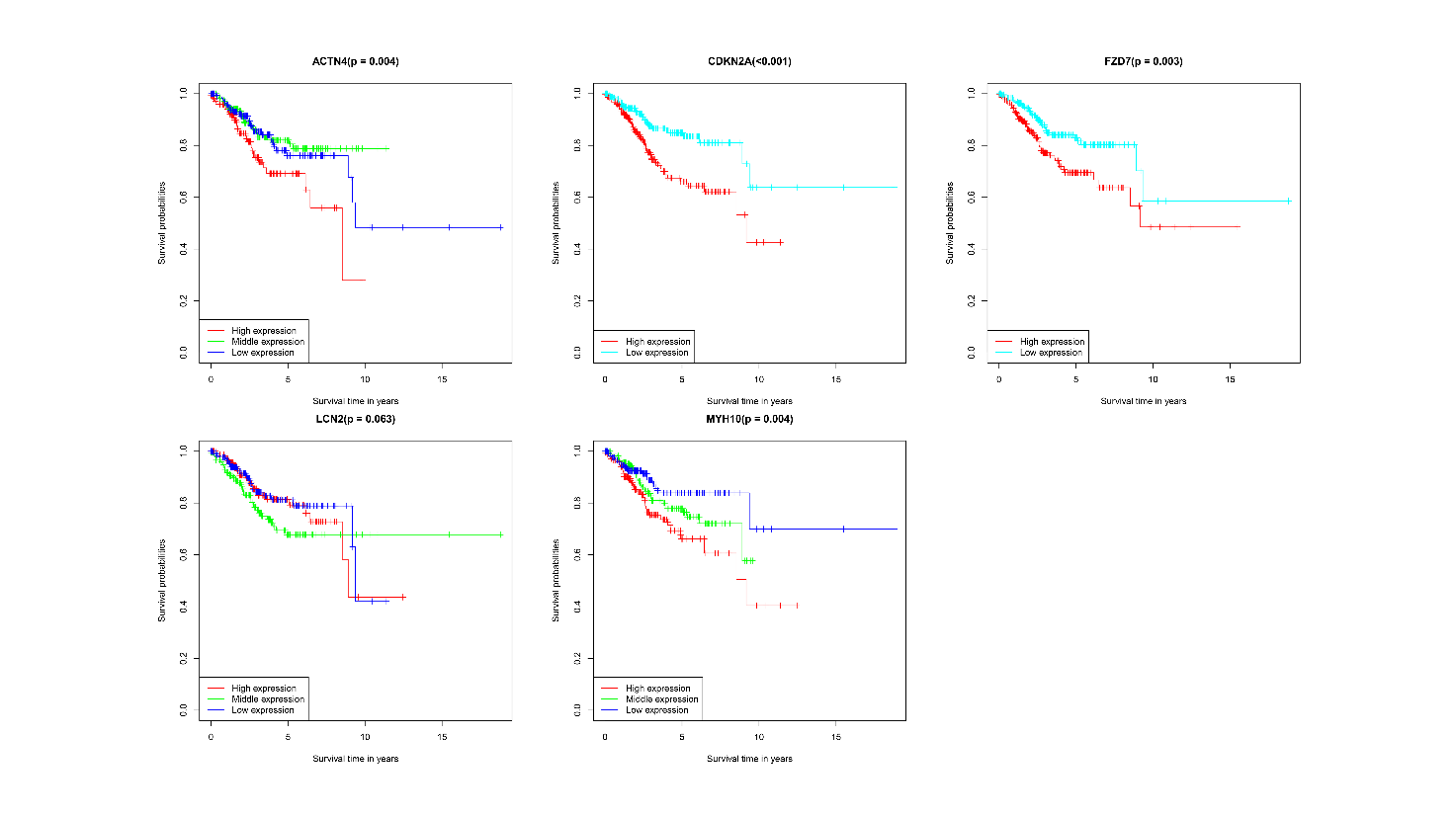


## Supplemental Figure 3: COX analysis was used to verify miRNAs related to UCEC patients


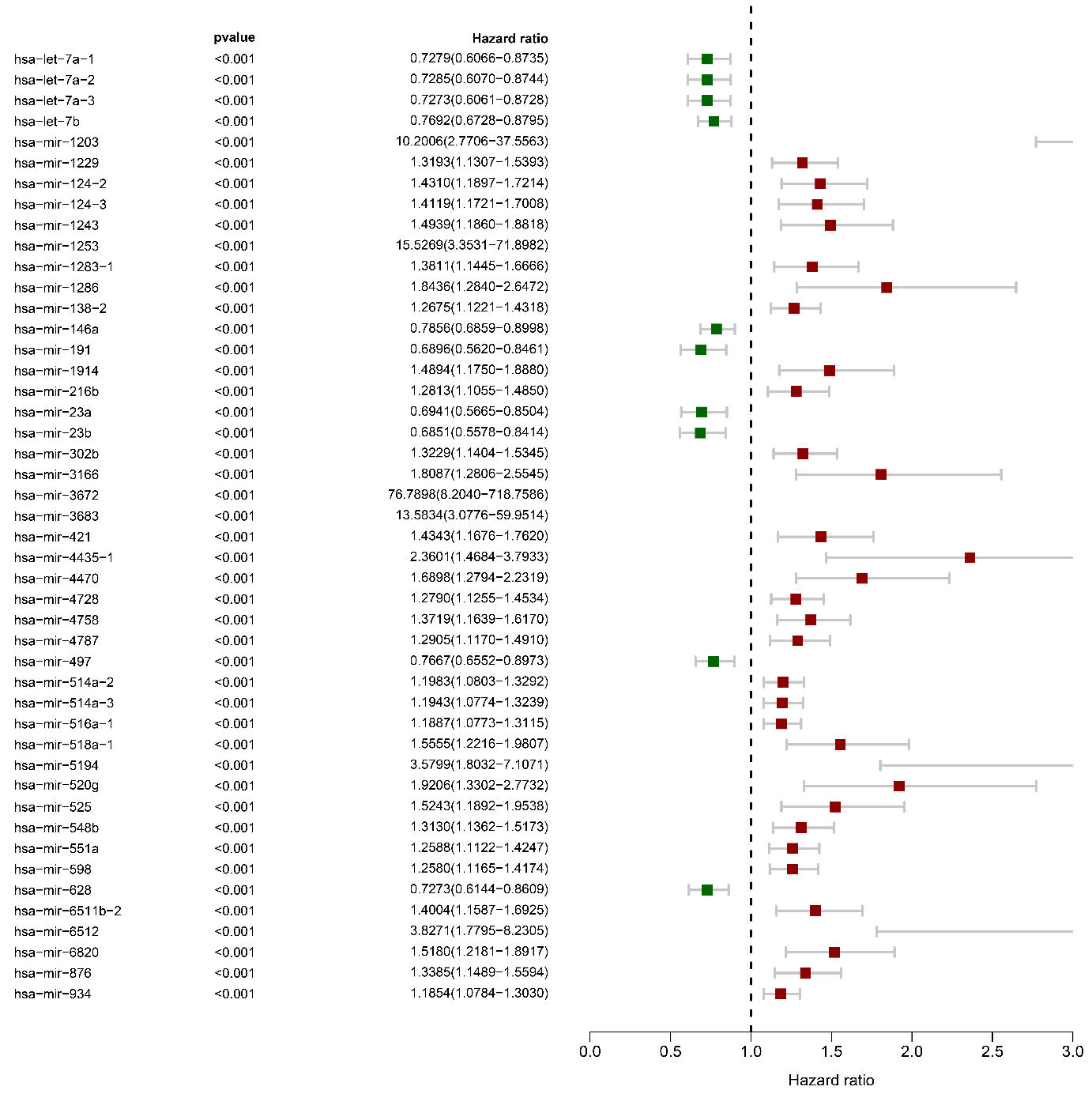

Supplement: Supplementary file 1 [file DataSheet1.docx]
